# Retrospective investigation of porcine circoviruses in cases of porcine dermatitis and nephropathy syndrome

**DOI:** 10.1186/s13567-024-01405-8

**Published:** 2024-11-09

**Authors:** Àlex Cobos, Mariano Domingo, Mónica Pérez, Eva Huerta, Anna Llorens, Joaquim Segalés, Marina Sibila

**Affiliations:** 1 Unitat Mixta d’Investigació IRTA-UAB en Sanitat Animal, Centre de Recerca en Sanitat Animal (CReSA), Campus de la Universitat Autònoma de Barcelona (UAB), 08193 Bellaterra, Barcelona Spain; 2 Departament de Sanitat i Anatomia Animals, Facultat de Veterinària, Campus de la Universitat Autònoma de Barcelona (UAB), 08193 Bellaterra, Barcelona Spain; 3 IRTA Programa de Sanitat Animal, Centre de Recerca en Sanitat Animal (CReSA), Campus de la Universitat Autònoma de Barcelona (UAB), 08193 Bellaterra, Barcelona Spain; 4 WOAH Collaborating Centre for the Research and Control of Emerging and Re-Emerging Swine Diseases in Europe (IRTA-CReSA), 08193 Bellaterra, Barcelona Spain

**Keywords:** Porcine dermatitis and nephropathy syndrome (PDNS), porcine circovirus 1 (PCV-1), porcine circovirus 2 (PCV-2), porcine circovirus 3 (PCV-3), porcine circovirus 4 (PCV-4), in situ hybridization, real-time quantitative polymerase chain reaction (qPCR)

## Abstract

Porcine dermatitis and nephropathy syndrome (PDNS) is a severe condition that affects mainly growing pigs and is considered to be caused by a type III hypersensitivity reaction. Although porcine circovirus 2 (PCV-2) is the antigen linked to this condition, porcine circovirus 3 (PCV-3) has also been proposed to be causally associated with PDNS. Moreover, the initial description of porcine circovirus 4 (PCV-4) also related this novel agent to this clinicopathological entity. Therefore, this retrospective study included a large number of PDNS cases (*n* = 102) fulfilling specific histologic criteria in search of known porcine circoviruses (PCV-1 to PCV-4) by conventional and/or quantitative PCR (qPCR). All the samples were subjected to PCV-2 immunohistochemistry (IHC) or conventional in situ hybridization (C-ISH), and RNAscope^®^ (R-ISH) was used to study PCV-2 and PCV-3 localization in a subset of the samples. All PDNS cases were PCV-2 positive by qPCR, while 30 of them (29.4%) yielded PCV-3 qPCR positivity; PCV-2 viral loads were significantly greater than PCV-3 viral loads. All animals were negative for PCV-1 and PCV-4. By C-ISH/IHC, 63 cases (61.8%) were positive for PCV-2, with low to moderate amounts of antigen. R-ISH demonstrated higher sensitivity, as all studied cases were positive; however, neither PCV-2 nor PCV-3 were consistently found within characteristic PDNS lesions. These results indicate that all PDNS-affected pigs were infected with PCV-2, emphasizing the likelihood that this viral antigen is causally linked to this condition. In contrast, no evidence of the association of PCV-1, PCV-3 or PCV-4 with PDNS was found.

**Supplementary Information:**

The online version contains supplementary material available at 10.1186/s13567-024-01405-8.

## Introduction

Porcine dermatitis and nephropathy syndrome (PDNS) is a clinically and pathologically well-defined condition that affects mainly growing pigs [1]. Clinically, affected pigs exhibit anorexia and depression without hyperthermia and develop irregular skin lesions that evolve from macules to papules to necrotic crusted lesions, which are mostly centered in the hind limbs and perianal area but may become generalized. Although this syndrome has high mortality rates ranging from 50 to 100%, it affects few pigs within a herd, with a prevalence rarely over 1% [2].

From a pathologic point of view, PDNS is an immune-mediated vascular disease resulting in necrotizing vasculitis, ischemia and haemorrhage in multiple organs. First, capillaries of the glomerular tufts are severely affected, producing fibrino-necrotizing glomerulitis with exudation of neutrophils and fibrin into Bowman’s space, a lesion that generally causes death by producing acute renal insufficiency. Eventually, if an animal survives acute disease, healing of the lesions leads to glomerulosclerosis and chronic interstitial nephritis [1]. Other typically affected vessels are dermal capillaries and arterioles and arteries in the mesentery and spleen [3], which show necrotizing and neutrophilic leukocytoclastic vasculitis. Vascular damage is believed to be caused by a type III hypersensitivity reaction based on the presence of immunoglobulin and complement components in damaged vessels and glomeruli. However, electron microscopy inconsistently revealed dense deposits in glomerular basement membranes across different studies [4, 5].

Since its first description in 1993 [6], various etiologies have been proposed as causative antigens that are able to trigger PDNS. In one study, *P. multocida* was isolated from 16 out of 20 studied pigs from tissues or body fluids [7]. This finding was supported by the further isolation of *P. multocida* strains showing a specific pulse‒field gel electrophoresis pattern from PDNS cases [8]. However, the bacterial etiology of PDNS was not further considered. In fact, the most investigated agents triggering PDNS are indeed viruses. Soon after its first description, case collections from Spain and Canada suggested a link with porcine reproductive and respiratory syndrome virus (PRRSV) on the basis of seropositivity and virus isolation from affected animals [1, 9]. This hypothesis was ruled out after diagnoses of PDNS in PRRSV-free herds, as were descriptions of PDNS cases in which PRRSV was specifically investigated and discarded [5]. Torque teno sus viruses (TTSuV) 1 and 2 were investigated in one study, which revealed a greater trend of TTSuV2 presence in the serum of PDNS-affected pigs than in that of healthy pigs, but the pathogenic potential of TTSuV2 in causing PDNS was considered negligible [10].

Despite previous suggestions, the most likely hypothesis has linked PCV-2 as the antigen triggering PDNS, since a number of studies have consistently reported PCV-2 seropositivity and viral presence within the tissues of affected pigs [11–16]. In fact, PDNS has long been considered a porcine circovirus disease (PCVD) [2, 3, 17]. However, compared with cases of PCV-2 systemic disease (formerly postweaning multisystemic wasting syndrome), those with PDNS consistently have lower serum viral loads [18], suggesting a different pathogenic mechanism, probably of an immune-mediated nature. Perhaps the most convincing evidence of causal associations is a case‒control study that revealed extremely high PCV-2 antibody titres in PDNS-affected animals compared with healthy animals, as well as increased accumulation of immunoglobulins (IgG1, IgG2 and IgM) and complement factors (C1q and C3) in renal glomeruli, further suggesting that PDNS is a type III hypersensitivity reaction to PCV-2 [19]. Importantly, although PCV-2 is consistently present in cases of PDNS, viral presence within tissues by ISH or IHC (if present) is restricted to lymphoid tissues, sparing affected vessels or glomeruli [17]. This absence of positivity in affected tissues has often been associated with suboptimal ISH and IHC sensitivity and/or clearing of antigens within the lesions. RNAscope^®^ in situ hybridization (R-ISH) is a new generation of ISH optimized to detect RNA/ssDNA with high sensitivity, with single copies of genetic material displayed as single dots [20]. To date, this technique has not been used for the detection of PCV-2 in PDNS cases.

In 2016, PCV-3 was discovered by next-generation sequencing methods in sows affected by PDNS [21] and was subsequently detected in cases of PDNS-affected pigs [22]. Indeed, PCV-3 is associated with the development of different types of vascular lesions, including lymphohistiocytic arteritis and periarteritis [23–25], which differ from those observed in PDNS cases (leukocytoclastic arteritis). In fact, a retrospective study on PCV-3-associated lesions demonstrated the presence of viral DNA in these abovementioned lesions, but in contrast, all 10 studied cases of PDNS yielded negative results [26]. Therefore, whether PCV-3 is eventually the antigen associated with PDNS cases is at least questionable.

Although many studies have investigated the presence of either PCV-2 or PCV-3 in cases of PDNS, none have thoroughly evaluated the presence of all PCVs. For example, PCV-2 was retrospectively tested in fewer than 30 PDNS cases [27] when PCV-3 was still undiscovered. PCV-3 was detected in 45/48 PDNS cases in a study in which PCV-2 was not specifically investigated [21]. In another study, 11/11 cases labelled as PDNS were positive for PCV-3 and negative for PCV-2, although fibrinonecrotizing glomerulonephritis was not observed [22]. Therefore, the present study aimed to retrospectively evaluate a high number of PDNS cases strictly diagnosed under its histological criteria for the presence of all known porcine circoviruses (PCV) to further elucidate their potential involvement in PDNS occurrence.

## Materials and methods

### Retrospective case selection

**Figure 1:**
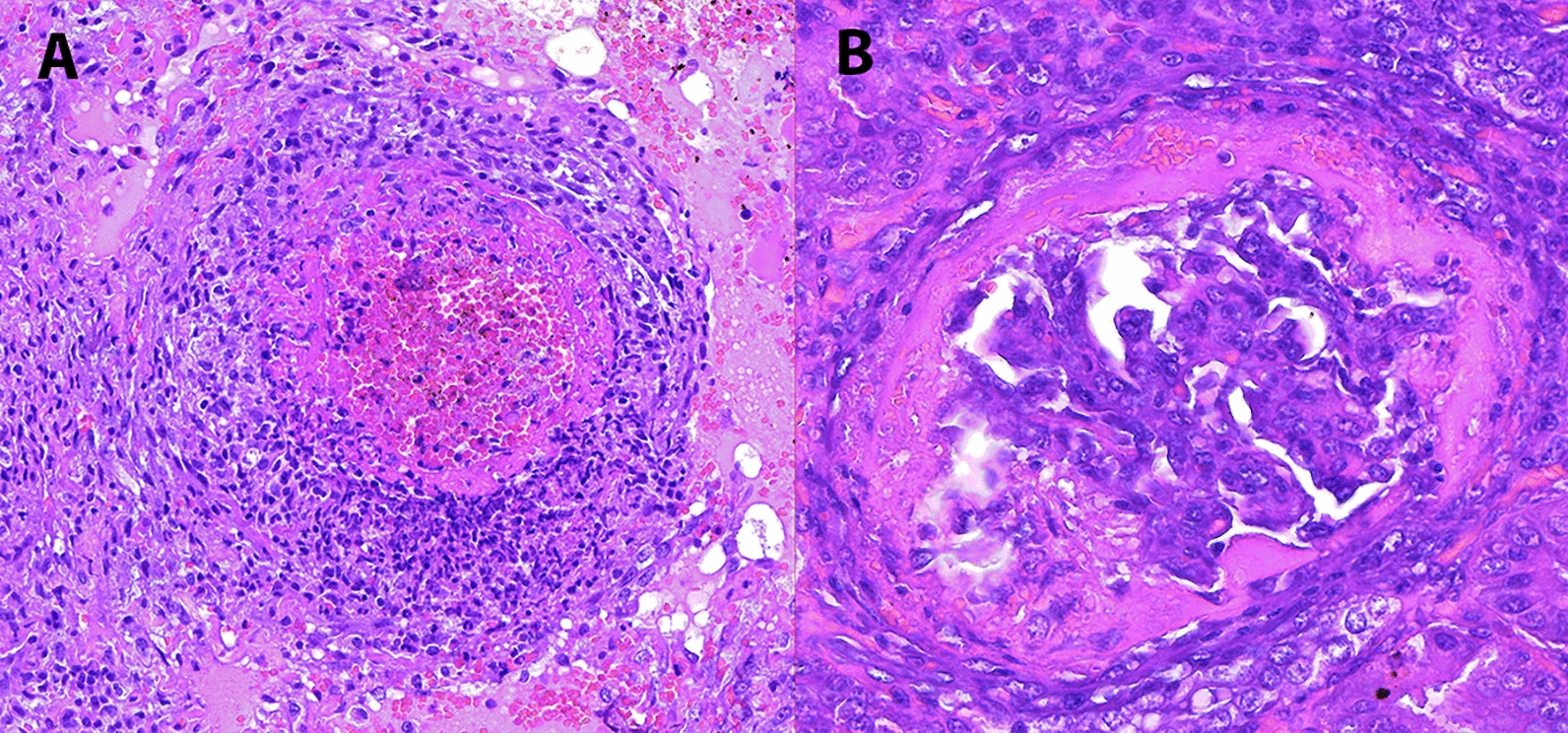
**Inclusion criteria for PDNS cases**. (**A**) Leukocytoclastic arteritis, characterized by fibrinoid necrosis and infiltration by leukocytes (mostly neutrophils) undergoing karyolysis and karyorrhexis. (**B**) Fibrinonecrotizing glomerulonephritis characterized by massive fibrin exudation into Bowmann’s space. Haematoxylin‒eosin (HE) staining.

This study was conducted on formalin-fixed, paraffin-embedded (FFPE) archived pig samples submitted to the *Servei de Diagnostic en Patologia Veterinaria* (SDPV) at the *Universitat Autònoma de Barcelona* (UAB). For the purpose of this study, the SDPV database was filtered to search for pigs diagnosed with PDNS on the basis of their clinical history and compatible skin and renal lesions. Only cases histologically confirmed with acute fibrinonecrotizing glomerulonephritis and systemic necrotizing vasculitis were included (Figure 1); samples from pigs with chronic lesions such as glomerulosclerosis were excluded. Tissues from all the animals (including at least one lymph node and kidney) were previously tested for PCV-2 by means of conventional in situ hybridization (C-ISH) or immunohistochemistry (IHC) [28].

### DNA extraction from FFPE material and PCV PCRs

The archived FFPE tissue blocks included at least the lymph nodes and kidneys. A total of four 8 μm tissue slices were cut using a microtome, placed in an Eppendorf tube and treated first with 1 mL of xylol three times and then with 1 mL of absolute ethanol three times. The obtained tissue pellet was subsequently dried and resuspended according to the instructions of the QiAamp^®^ DNA FFPE Tissue Kit. The DNA from these samples was extracted as described previously [23].

Genome presence of all known PCVs was investigated in extracted DNA by conventional PCR or real-time quantitative PCR (qPCR). For PCV-1, a conventional PCR was used as previously described [29]. For PCV-4, a conventional PCR was used to target the Rep gene using previously described primers [30]. Therefore, the results were expressed as positive or negative. For PCV-2 and PCV-3, specific qPCRs were used. For PCV-2, a commercially available qPCR method was used (LSI VetMax^tm^ Porcine Circovirus Type 2 Quantification, Thermo Fisher Scientific). For PCV-3, qPCR was performed as previously described [31]. For both qPCR methods, viral loads were expressed as PCV-2 or PCV-3 genome copies/mL of tissue supernatant. For PCV-2 and PCV-3 qPCR, results below 10^4^ and 10^3^ viral genome copies/mL of tissue supernatant were considered positive but below the quantification limit (BQL), respectively. In those cases, the limit of quantification divided by 2 was used as the numeric result [32]. Cases were classified according to their quantifiable viral load for either PCV-2 or PCV-3 as follows: medium viral load (<10^6^ genome copies/mL of tissue supernatant) and high viral load (≥-10^6^ genome copies/mL of tissue supernatant).

### PCV-2 and PCV-3 in situ hybridization (R-ISH)

To locate the PCV-2 and PCV-3 genomes within tissues, an ISH assay using RNAscope^®^ technology (R-ISH) was performed on a subset of samples. For PCV-2 genome localization, a probe targeting the PCV-2 genome (catalogue no. 491018, RNAscope^®^) was used, following the manufacturer’s instructions. For PCV-3, a probe targeting ORF1 was used as previously described [31]. Lymph nodes positive for either PCV-2 or PCV-3 were used as positive controls. A lymph node negative for all circoviruses was used as a negative control. The presence and distribution of the viral genome within lymphoid tissues were recorded and scored following a previously described system for PCV-2 [28]: a score of 1 indicated the presence of the viral genome only in follicular areas; a score of 2 also included the presence of few labelled cells in parafollicular areas, whereas a score of 3 indicated a generalized distribution of the virus in both the follicular and parafollicular areas. For renal tissue, similar scores were recorded according to the amount of labelling as (1) mild, (2) moderate or (3) abundant within the renal parenchyma and inflammatory infiltrates.

### Statistical analyses

All the graphics and statistical analyses were generated with GraphPad Prism 10.1.2 software. Viral load differences were analysed through the Mann‒Whitney test. Only statistically significant differences are represented in the graphics, with the *p* value being represented in the graphic. Statistical significance was set at *p* < 0.05.

## Results

### Retrospectively selected cases

**Figure 2:**
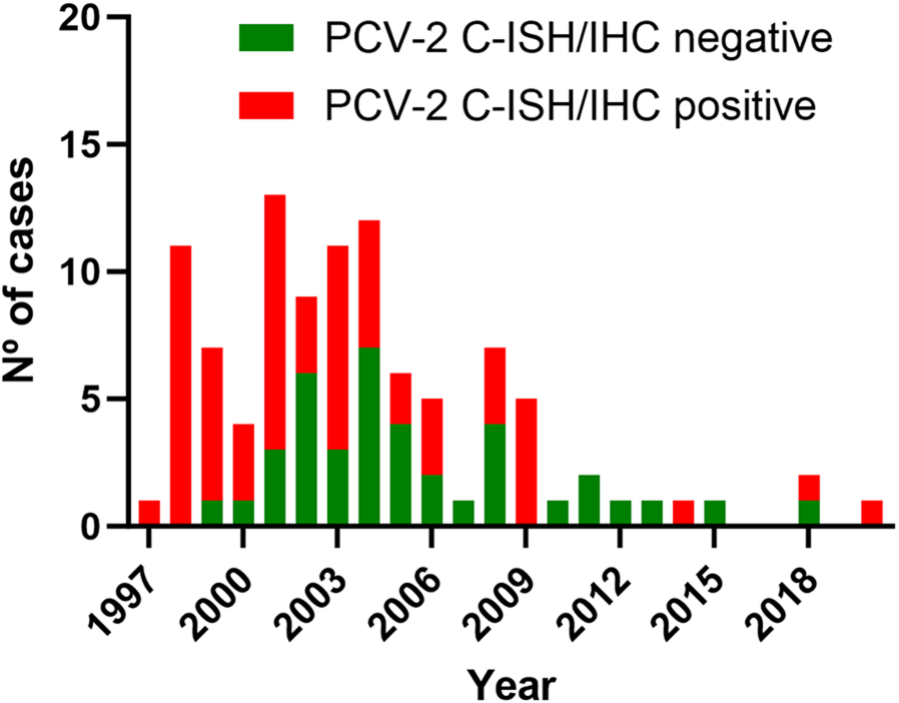
**Number of PDNS cases per year and their results for PCV-2 presence by C-ISH or IHC.**

The 102 cases included in this study, who fulfilled the criteria for acute PDNS, were received between 1997 and 2020. Among them, 39 had negative results against PCV-2 by either C-ISH or IHC, whereas the remaining 63 had positive results, usually with low to moderate amounts of genome or antigen located mainly in the lymph nodes. The number of selected cases per year and their PCV-2 status by IHC or C-ISH are available in Figure 2. Individual results are provided in Additional file 1.

### PCR and qPCR results

All assessed cases were negative by means of conventional PCRs against PCV-1 and PCV-4.

**Figure 3:**
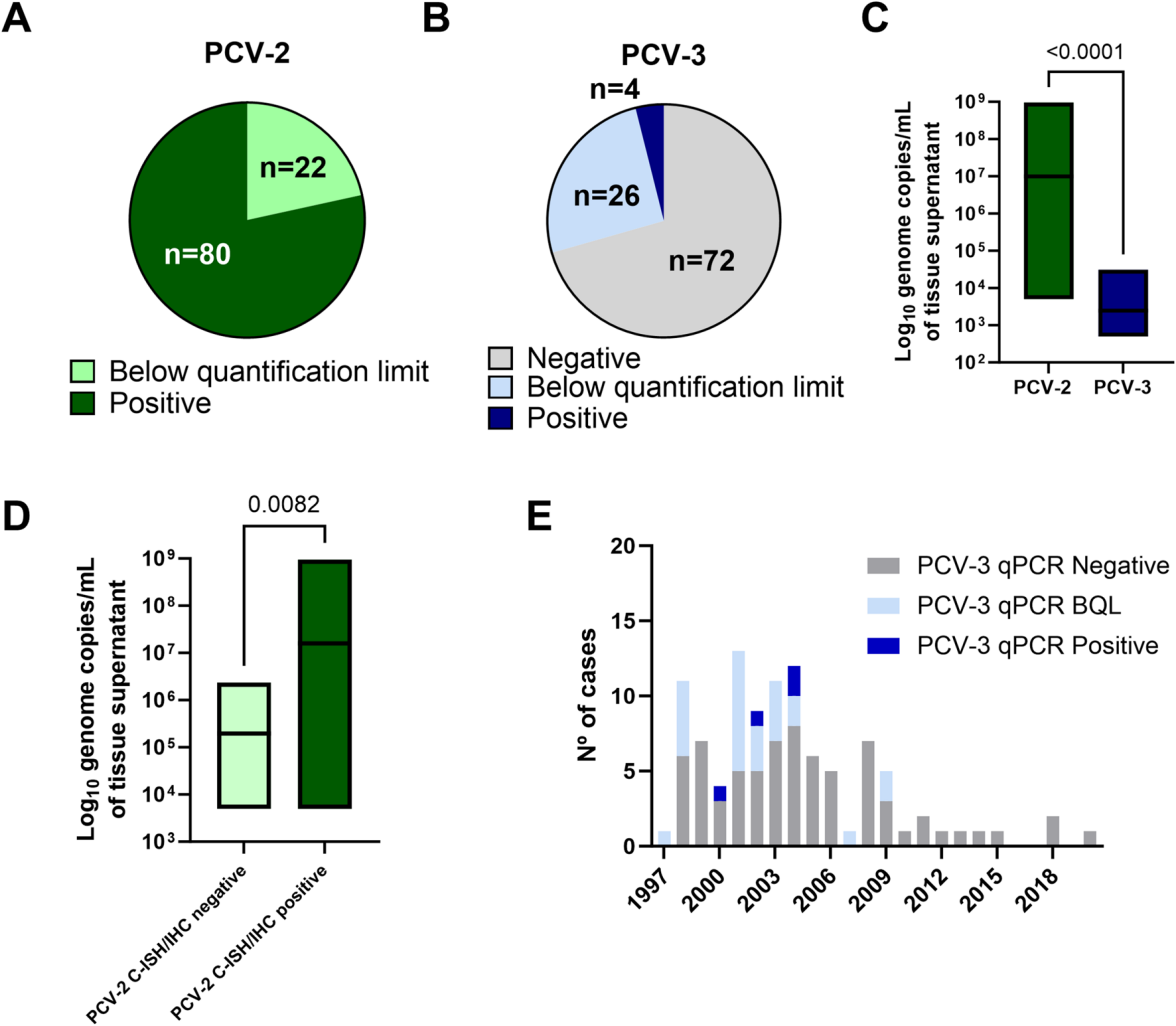
**qPCR results for PCV-2 and PCV-3.** **A** Number of cases with positive results and those below the quantification limit according to PCV-2 qPCR. **B** Number of cases that were negative, below the quantification limit and positive according to PCV-3 qPCR. **C** Boxplot of PCV-2 and PCV-3 qPCR results (line at the mean). **D** Boxplot of PCV-2 qPCR results, segregated by C-ISH/IHC-positive and -negative cases (line at the mean). **E** Distribution of positive, BQL and negative cases against PCV-3 by qPCR in the studied years.

All investigated cases (102/102, 100%) were positive for PCV-2 through qPCR, with 22/102 (21.7%) having a viral load below the quantification limit (Figure 3A). On the other hand, 30/102 (29.4%) cases were qPCR positive for PCV-3, with 26/102 (25.5%) being below the quantification limit (Figure 3B). The average viral load for PCV-2 was significantly greater (9.88 × 10^6^ genome copies per mL of tissue supernatant, ranging from 1.03 × 10^4^–9.49 × 10^8^ genome copies per mL of tissue supernatant) than that of PCV-3 (average 2.47 × 10^3^ genome copies per mL of tissue supernatant; ranging from 1.93 × 10^3^–3.08 × 10^4^ genome copies per mL of tissue supernatant) (Figure 3C). When segregated by their C-ISH/IHC results, the C-ISH/IHC PCV-2-positive cases had significantly greater viral loads than did the C-ISH/IHC-negative cases (Figure 3D). PCV-3-positive cases were found in almost every analysed year (Figure 3E). Individual results by case are available in Additional file 1.

### PCV-2 and PCV-3 R-ISH results

PCV-2 R-ISH was performed in 5 cases with PCV-2 qPCR results below the quantification limit, 10 cases with medium PCV-2 loads, and 10 cases with high PCV-2 loads. PCV-3 R-ISH was performed in 5 cases below the quantification limit (for PCV-3) and the 4 quantifiable cases (which had medium PCV-3 loads). The cases selected for R-ISH and their qPCR results are available in Additional file 2.

**Figure 4:**
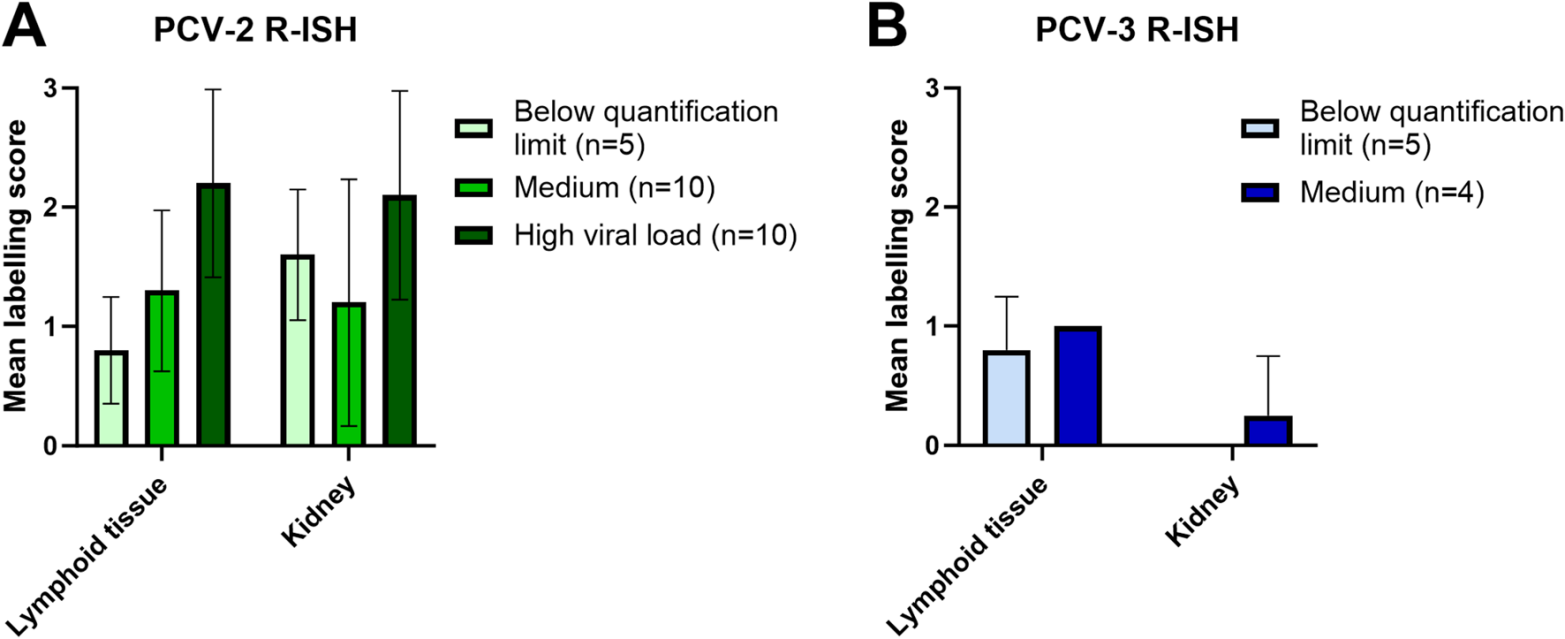
**R-ISH results for PCV-2 and PCV-3.** **A** Mean labelling score by R-ISH against PCV-2 in the lymphoid tissues and kidneys of all studied cases classified by their viral loads by qPCR in pooled tissues. **B** Mean labelling score by R-ISH against PCV-3 in all lymphoid tissues and kidneys of the studied cases classified by their viral loads by qPCR in pooled tissues.

PCV-2 labelling was observed in all 25/25 analysed cases, either in lymphoid tissues (in 23/25 tested cases, mean score of 1.56), mainly in follicular areas, or in renal tissue (in 21/25 cases, mean score of 1.64), within inflammatory infiltrates and tubular epithelial cells. PCV-3 nucleic acids were also consistently present within lymphoid tissues in 9/9 studied cases (mean score 0.89); in renal tissue, only one case was labelled (Figure 4). The R-ISH scores for each case and their correlations with the qPCR and C-ISH/IHC results are available in Additional file 2.

**Figure 5:**
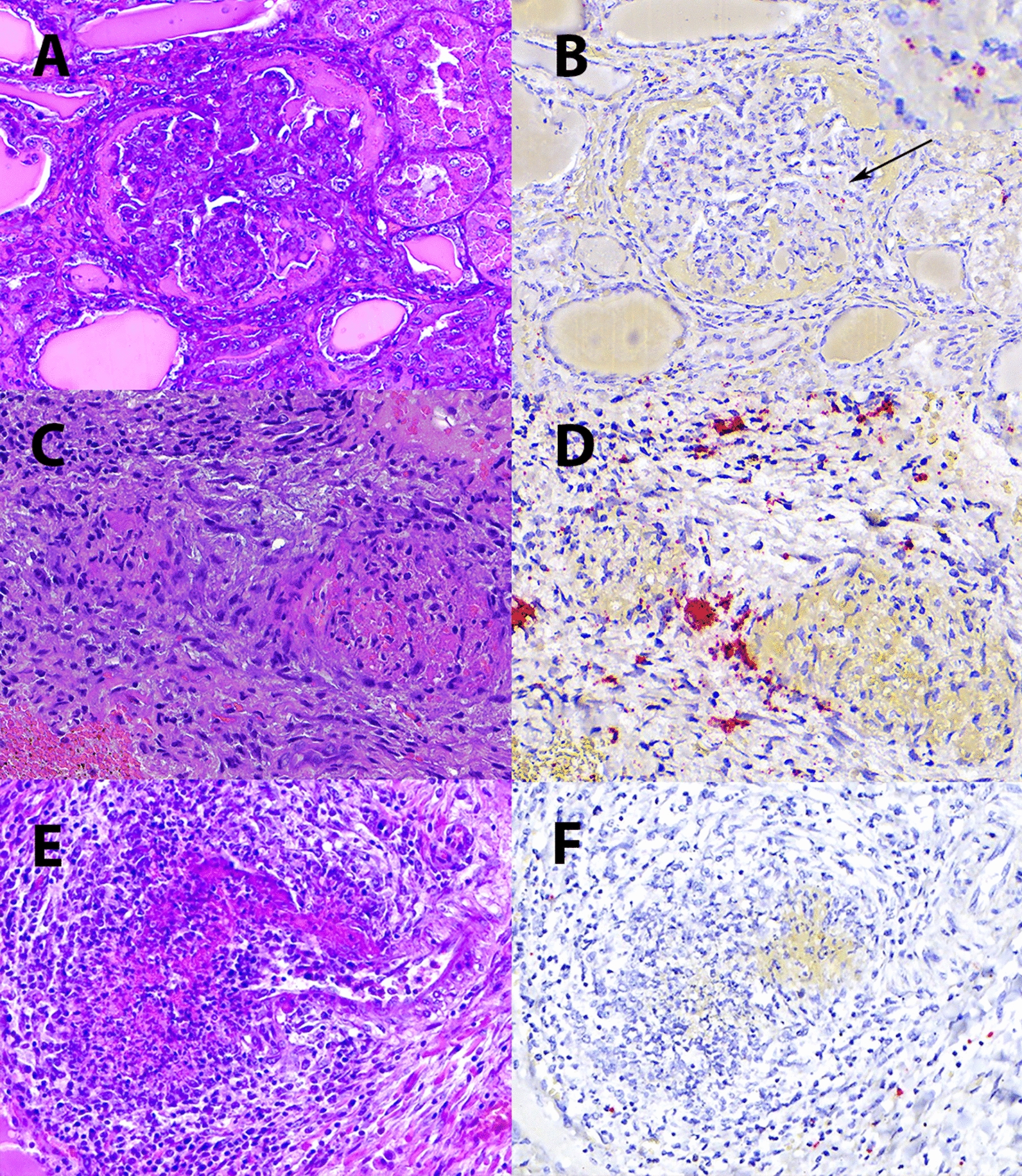
**R-ISH against PCV-2.** **A** Glomeruli affected by fibrinonecrotizing glomerulonephritis. **B** Presence of very scant PCV-2 signal (red stain) in the glomerular capillaries (arrow). **C** Leukocytoclastic arteritis in the renal pelvis. **D** Positive PCV-2 signal (red stain) in the endothelial and muscular cells within the affected artery. A strong positive signal was observed in inflammatory cells in the tunica adventitia. **E** Renal artery showing segmental leukocytoclastic vasculitis. **F** Complete absence of PCV-2 signals within the vascular lumen, endothelium and leukocytoclastic infiltrate. Haematoxylin‒eosin staining (**A**, **C**, **E**) and PCV-2 R-ISH (**B**, **D**, **F**).

For the detection of PCV-2 within fibrinonecrotizing glomerulonephritis, in 13/25 cases (52.0%), single dots were focally and segmentally detected in very few affected glomeruli, leaving most of the glomeruli with total absence of staining (Figures 5A and B). Similarly, 14/25 cases (56.0%) displayed some degree of labelling within arteries and arterioles (regardless of the presence of leukocytoclastic arteritis) (Figures 5C and D); however, most of the affected arteries displayed a complete absence of viral signals (Figures 5E and F).

**Figure 6:**
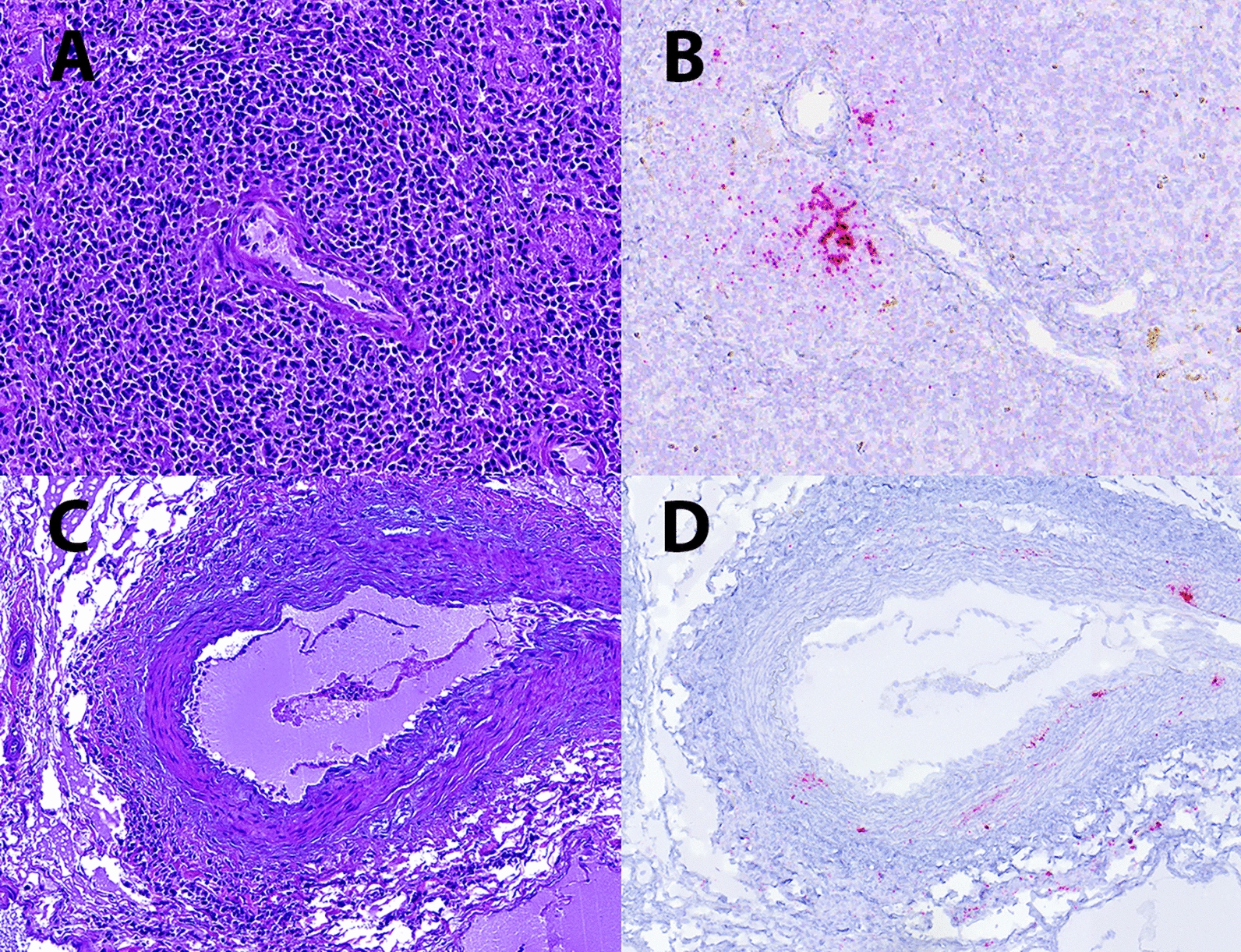
**R-ISH against PCV-3.** **A** Normal periarteriolar lymphoid sheath (PALS) in the spleen. **B** Presence of a positive PCV-3 signal (red stain) in the same PALS by ISH against PCV-3. **C** Presence of lymphohistiocytic periarteritis in a medium calibre artery in the renal pelvis. **D** Positive PCV-3 signal (red stain) in the tunica media and in inflammatory cells in the tunica adventitia of the same artery. Haematoxylin‒eosin staining (**A**, **C**) and PCV-3 R-ISH (**B**, **D**).

Among the nine cases assessed, the PCV-3 genome was located mainly in the germinal centres of lymphoid tissues (Figure 6A and B). Only in one case, viral nucleic acid was observed in the tunica media of a medium-sized artery in the renal pelvis, which was affected by lymphohistiocytic (but not necrotizing) periarteritis (Figure 6C and D). No labelling was observed in glomeruli affected by fibrinonecrotizing glomerulonephritis or in arteries affected by leukocytoclastic arteritis. In addition, labelling was not observed in renal tubules or interstitial infiltrates.

## Discussion

The present study further expands the existing knowledge on PCVDs, specifically the PDNS. A large number (*n* = 102) of cases fulfilling the histopathological diagnostic criteria (fibrinonecrotizing glomerulonephritis and leukocytoclastic arteritis) were investigated for the presence of all known porcine circoviruses.

PCV-2 is among the most impactful pathogens in the swine industry and is capable of producing a variety of well-recognized diseases. Although it is included as a PCVD, PDNS is considered an immune-mediated disease [19], and its association with PCV-2 has been circumstantial, mainly because of the high PCV-2 antibody titres, which are believed to trigger a type III hypersensitivity reaction. Moreover, the overall number of PDNS cases seems to have greatly decreased since the widespread utilization of PCV-2 vaccines, i.e., approximately 2008 [33], and this decrease was also evident in our study. PDNS pathogenesis involves the deposition of immune complexes, the activation of complement and the development of leukocytoclastic vasculitis in the renal glomeruli and arteries. Clear identification of the antigen triggering PDNS and hence its causal agent was lacking, which is an obstacle to definitive causal association.

PCV-3 was first associated with pigs with a PDNS-like condition [21], followed by multiple descriptions of PDNS-like disease [22, 35]. Moreover, an experimental infection with a PCV-3 infectious clone was claimed to reproduce PDNS-like lesions, although fibrinonecrotizing glomerulonephritis and leukocytoclastic arteritis were not generated [34]. More recent descriptions of naturally and experimentally occurring disease have further characterized the outcome of PCV-3 infection, with the most characteristic lesions being lymphohistiocytic arteritis and periarteritis [26, 36], which differ from PDNS characteristic histologic lesions.

The present study failed to demonstrate regular PCV-3 presence in cases of PDNS, as only 29.4% of the cases were positive for PCV-3 by qPCR, in contrast to all the cases that were positive for PCV-2. Moreover, the PCV-3 loads were consistently lower than those of PCV-2. In fact, both PCV-2 and PCV-3 circulate endemically to a moderate prevalence in intensively reared swine farms. In a study targeting European farms, whereas PCV-2 was detected in 21% and 47% of the samples and farms assessed, respectively, the PCV-3 prevalence ranged from 4.0 to 59.5% in healthy pigs [37]. Taking this into account, the positivity for PCV-3 in 29.4% of the samples in our study probably reflects the expected endemic circulation; in contrast, 100% of the samples positive for PCV-2 by qPCR strongly suggest the involvement of this virus in PDNS causality. There are also previous studies that failed to statistically correlate PCV-3 presence with PDNS occurrence [38].

To further support this association, identification of the antigen triggering the hypersensitivity response should be mandatory. To date, no antigen has been detected within PDNS characteristic lesions across different studies, which impedes the definitive establishment of causality. A study using a double-labelling technique identified PRRSV and PCV-2 in PDNS cases [39]; however, PCV-2 was inconsistently found in leukocytoclastic arteritis and fibrinonecrotizing glomerulonephritis, and it was proposed that those antigens would have already been cleared from the necrotizing lesions by the elicited inflammatory reaction or that techniques used to evaluate the presence of these viruses were not sensitive enough. Therefore, a more sensitive technique able to detect the pathogen within lesions would be beneficial for assessing potential lesion causality. Although different viral components (protein through IHC and genome by C-ISH) are detected, both techniques are suitable for diagnostic purposes, as they have similar performance in tissues with a certain viral load [40]. In contrast, RNAscope^®^ technology has the potential to detect single copies of single-stranded nucleic acids as individual red dots [20]. In our study, RNAscope^®^ technology indeed displayed superior sensitivity, as all the selected cases (25/25, 100%) were positive according to R-ISH, with 7 out of those 25 cases being negative according to C-ISH/IHC. However, if PDNS was caused by immunocomplexes formed by viral particles of PCV-2 (carrying copies of ssDNA) and antibodies, one would expect the regular presence of labelling across damaged glomeruli and arteries in PDNS cases, but this was not observed in our study or in any other study. Hence, we hypothesize that the pathogenesis of PDNS might involve (a) immunocomplexes formed by PCV-2 proteins and not full viral particles (type III hypersensitivity reaction) or (b) the formation of autoantibodies against endogenous proteins of vessels, such as the basement membrane (type II hypersensitivity reaction). In human medicine, several autoimmune vasculopathies (e.g., Goodpasture syndrome and ANCA-associated vasculitis) related to this latter pathogenic mechanism show significant similarities with PDNS from a pathological point of view [41, 42]. For example, Goodpasture syndrome is caused by antibodies against vascular basement membrane proteins, which affect glomeruli and alveolar capillaries. Interestingly, IgG has also been previously detected by IHC in the pulmonary tissues of PDNS cases [19]. However, it is not possible to rule out, as previously mentioned, that the PCV-2 genome could have been removed from lesions by the elicited inflammatory reaction.

On the other hand, the studied cases were positive through R-ISH against PCV-3, with the presence of PCV-3 being largely confined to lymphoid tissues. In only one of the 9 tested cases, the PCV-3 genome was detected in a medium-sized muscular artery displaying lymphohistiocytic periarteritis, but PDNS lesions were negative for the presence of the PCV-3 genome. This suggests that PDNS and PCV-3-associated lesions were concomitant in this case but does not indicate potential causality of PDNS by PCV-3. Although both PDNS and PCV-3-associated diseases appear to be vascular disorders in terms of their histopathological observations, their associated lesions have different natures (leukocytoclastic vs. lymphohistiocytic). For this reason, precise histologic evaluation and description can be reliably used to discriminate these vascular diseases.

All assessed PDNS cases in this study were negative for PCV-1 and PCV-4. Although PCV-1 was the first discovered porcine circovirus, it is known to subclinically circulate with limited prevalence in swine herds [43]. On the other hand, PCV-4 is the latest discovered PCV-4, first detected in pigs from China with respiratory disease, diarrhoea and skin lesions suggestive of PDNS [44]. However, most studies failed to detect PCV-4 in Europe [45] until a recent study revealed positive wild boar and Iberian pigs in mid-southwestern Spain [30]. Overall, on the basis of the obtained results, the potential of these two circoviruses in causing PDNS seems largely unlikely.

In summary, this study further highlights the notion that, among all known PCVs, PCV-2 is likely the pathogen related to PDNS triggering, since it was detected in all tested cases by sensitive techniques (qPCR and R-ISH). PCV-3 was also detected in some PDNS cases but at a much lower frequency, suggesting normal viral circulation rates in swine. Finally, no evidence of PCV-1 or PCV-4 infection was found in the studied PDNS cases.

## Supplementary Information

**Additional file 1. qPCR, R-ISH, C-ISH and IHC results per case.**

**Additional file 2. Summary of 25 cases selected for PCV-2 and PCV-3 DNA localization by R-ISH and their qPCR results.**

## Data Availability

All the data used in this review are derived from diagnostic cases that are pertinently archived (available but not public data), and some data can be consulted on the supplementary data attached.
