# Supplementary material for: Retrospective investigation of porcine circoviruses in cases of porcine dermatitis and nephropathy syndrome
Source: Vet Res. 2024 Nov 9;55:146. doi: 10.1186/s13567-024-01405-8 (PMC11549871; doi:10.1186/s13567-024-01405-8)
Supplement: Supplementary file 2 — Additional file 2. Summary of 25 cases selected for PCV-2 and PCV-3 DNA localization by R-ISH and their qPCR results. [file 13567_2024_1405_MOESM2_ESM.docx]

|  |  |  |  |  | PCV-2 R-ISH | | PCV-3 R-ISH | |
| --- | --- | --- | --- | --- | --- | --- | --- | --- |
| Case ID | Year of submission | PCV-2C-ISH/IHC | PCV-2 viral load* | PCV-3 viral load* | Ly | Ki | Ly | Ki |
| 3022 | 2000 | - | 5.0E+3 (BQL) | 2.03E+04 | 0 | 2 | 1 | 0 |
| 2188 | 2002 | - | 5.0E+3 | 5.0E+02 | 1 | 2 | 0 | 0 |
| 3325 | 2002 | - | 5.0E+3 | - | 1 | 1 | NP | |
| 3119 | 2001 | + | 5.0E+3 | 5.0E+02 | 1 | 2 | 1 | 0 |
| 543 | 2003 | + | 5.0E+3 | - | 1 | 1 | NP | |
| 639 | 2005 | - | 1.26E+04 | - | 0 | 1 | NP | |
| 2200 | 1998 | + | 2.24E+04 | - | 2 | 1 | NP | |
| 1549 | 2004 | + | 6.00E+04 | 8.20E+03 | 2 | 1 | 1 | 1 |
| 1315 | 2002 | + | 8.41E+04 | 5.0E+02 | 2 | 2 | NP | |
| 1189 | 2005 | + | 9.90E+04 | - | 2 | 2 | NP | |
| 1626 | 2004 | - | 1.45E+05 | 1.93E+03 | 1 | 2 | 1 | 0 |
| 547 | 2008 | + | 2,05E+05 | - | 1 | 3 | NP | |
| 1533 | 2009 | + | 3,75E+05 | - | 1 | 0 | NP | |
| 479 | 2007 | - | 4,06E+05 | 5.0E+02 | 1 | 0 | 1 | 0 |

# **Additional file 2**. **Summary of 25 cases selected for PCV-2 and PCV-3 DNA localization by R-ISH and their qPCR results.**

| 9 | 2006 | + | 4,61E+05 | - | 1 | 0 | NP | |
| --- | --- | --- | --- | --- | --- | --- | --- | --- |
| 3373 | 2001 | + | 1.02E+06 | - | 3 | 2 | NP | |
| 2100 | 1998 | + | 1.05E+06 | 5.0E+02 | 2 | 2 | NP | |
| 605 | 2004 | + | 2.02E+06 | - | 2 | 2 | NP | |
| 645 | 2005 | - | 3.36E+06 | - | 1 | 3 | NP | |
| 336 | 2018 | + | 3.38E+06 | - | 3 | 2 | NP | |
| 586 | 2001 | + | 3.76E+06 | 5.0E+02 | 3 | 3 | 1 | 0 |
| 513 | 2002 | + | 4,22E+06 | 3.08E+04 | 2 | 0 | 1 | 0 |
| 2433 | 2001 | + | 5.30E+06 | 5.0E+02 | 1 | 2 | NP | |
| 822 | 1997 | + | 9.53E+06 | 5.0E+02 | 2 | 2 | 1 | 0 |
| 1127 | 1999 | + | 9,49E+08 | - | 3 | 3 | NP | |

# *Results are expressed as genome copies/mL of tissue supernatant. Ly: lymphoid tissues; Ki: kidney; NP: Not performed.
